# Personal medical electronic devices and walk-through metal detector security systems: assessing electromagnetic interference effects

**DOI:** 10.1186/s12938-017-0328-9

**Published:** 2017-03-20

**Authors:** Joshua Guag, Bisrat Addissie, Donald Witters

**Affiliations:** 0000 0001 2243 3366 grid.417587.8 U.S. Food and Drug Administration, 10903 New Hampshire Avenue, White Oak Building 62 Room 1131, Silver Spring, MD 20993 USA

**Keywords:** Medical device, Personal medical electronic devices, Electromagnetic interference, EMI, Electromagnetic compatibility, EMC, Security system, Metal detector, Walk through metal detector

## Abstract

**Background:**

There have been concerns that Electromagnetic security systems such as walk-through metal detectors (WTMDs) can potentially cause electromagnetic interference (EMI) in certain active medical devices including implantable cardiac pacemakers and implantable neurostimulators. Incidents of EMI between WTMDs and active medical devices also known as personal medical electronic devices (PMED) continue to be reported. This paper reports on emission measurements of sample WTMDs and testing of 20 PMEDs in a WTMD simulation system.

**Methods:**

Magnetic fields from sample WTMD systems were characterized for emissions and exposure of certain PMEDs. A WTMD simulator system designed and evaluated by FDA in previous studies was used to mimic the PMED exposures to the waveform from sample WTMDs. The simulation system allows for controlled PMED exposure enabling careful study with adjustable magnetic field strengths and exposure duration, and provides flexibility for PMED exposure at elevated levels in order to study EMI effects on the PMED. The PMED samples consisted of six implantable cardiac pacemakers, six implantable cardioverter defibrillators (ICD), five implantable neurostimulators, and three insulin pumps. Each PMED was exposed in the simulator to the sample WTMD waveforms using methods based on appropriate consensus test standards for each of the device type.

**Results:**

Testing the sample PMEDs using the WTMD simulator revealed EMI effects on two implantable pacemakers and one implantable neurostimulator for exposure field strength comparable to actual WTMD field strength. The observed effects were transient and the PMEDs returned to pre-exposure operation within a few seconds after removal from the simulated WTMD exposure fields. No EMI was observed for the sample ICDs or insulin pumps.

**Conclusion:**

The findings are consistent with earlier studies where certain sample PMEDs exhibited EMI effects. Clinical implications were not addressed in this study. Additional studies are needed to evaluate potential PMED EMI susceptibilities over a broader range of security systems.

## Background

This paper describes magnetic field emission measurement from walk-through metal detector (WTMD) and electromagnetic compatibility (EMC) testing performed on active medical devices for electromagnetic emission from WTMD. A number of different technologies such as metal detectors and electronic article surveillance (EAS, also known as anti-theft) systems are often referred to as security systems. These security systems use electromagnetic fields for detection of an object of metal and merchandise (EAS using special tags attached). Metal detector systems detects disturbance of the emitted low frequency magnetic field from the WTMD. The EAS systems detect passive or active tags on objects such as merchandise through various technologies that are differ from metal detectors in waveform and functions. At the lower frequency range, both security systems typically use near-field magnetic fields where these fields dominate the concerns for interference for the active medical devices. EAS systems operate in a wide range of frequencies from 20 Hz to 2.5 GHz [1]. Metal detectors typically operate at lower frequencies: 100 Hz to 10 kHz for WTMD and from 18 kHz to 1.8 MHz for hand-held metal detectors (HHMD) [2–6]. In normal operation these security systems typically involve short exposure times [1] to the emissions from the system, but can have a range of exposure amplitudes depending on the orientation and location near the emitters.

A number of studies in literature have reported or investigated potential electromagnetic interference (EMI) of active medical devices from electromagnetic (EM) sources such as cellular phone, TETRA radio transmitter, RFID, medical equipment (e.g., MRI, electrosurgery device, diathermy), and industrial equipment (e.g., arc welding, electric motor) [1, 3, 4, 7–11]. Among these EM sources, there have been specific concerns involving certain active medical devices such as implantable cardiac pacemakers and implantable neurostimulator (e.g., implanted spinal cord stimulators) being potentially vulnerable to EMI in proximity to security systems [1, 5, 6, 11]. There have been a few studies focus on metal detectors and active medical devices [5, 6, 12]. The Food and Drug Administration (FDA) recognized the issues associated with security systems and provided recommendations for patients with implantable medical devices [13]. There is also a published standard practice in checkpoint metal detector screening for patients with active implantable medical devices [14]. Medical device manufacturers acknowledged those issues as well and responded to reduce risks associated with potential EMI [4]. However, even with the publications and notices by the medical device manufacturers, there continue to be incident reports involving active medical devices and security systems. A recent search of reports in the FDA’s manufacturer and user facility device experience (MAUDE) database reveals there were more than 350 incident reports between 2014 and 2016 for certain active medical devices that appear to be related to metal detectors and security systems [15]. From such evidence there appears to be continuing issues involving EMI via exposure of active medical devices, also known as personal medical electronic devices or PMEDs, with the security systems.

Previous studies have evaluated possible interference between active medical devices and WTMD [5, 6, 12]. The studies done by Kainz et al. [5] and Seidman et al. [6] focus on implantable cardiac pacemaker and implantable neurostimulators using controlled in vitro testing for EMC and reveal potential EMI from exposure to WTMD. However, a study by Kolb et al. [12] suggests metal detectors have low probability to cause EMI on active medical devices. Because these previous studies were performed a number of years ago and included older generations of active medical devices, there is need to reassess potential EMI between newer generations of medical devices and security systems. More recently, Tiikkaja et al. conducted in vitro testing using explanted or demo implantable cardiac devices to study EMC for low frequency magnetic fields [16, 17]. In these studies, each medical device was exposed to low frequency magnetic fields from 2 Hz to 1 kHz using different waveforms such as sinusoidal, pulse, ramp and square waveforms. These studies provide valuable information that could eventually be used for standardized test method for low frequency magnetic field emitters. However as Tiikkaja et al. noted, the exposure waveform is an important parameter in assessing EMC with active implantable medical devices. As noted in our study the waveforms emitted by the WTMDs can change across spatial volume, and this makes it difficult to correlate generalized waveforms to exposure by actual WTMDs.

The purpose of the study herein is to expand in vitro testing performed in [5, 6] and evaluate the EMC of more recent models of higher risk devices that for the purpose of this study will be referred to as PMED. These devices deliver different therapies range from electrical stimulations to drug biological therapy. The study included characterization of sample WTMD used as the basis for recreating the exposure in the simulator system and allowing a broader range of exposures. The flexibility in controlled exposure via the simulator system aides in assessing the device effects related to PMED EMI. The study includes the following selected medical devices: implantable pacemakers, implantable cardioverter defibrillators (ICD), implantable neurostimulators, and insulin pumps. These medical devices are selected to represent PMEDs that are potentially susceptible to EMI from the WTMD [13]. Those devices were marketed devices at the time of the testing.

## Methods

The study was conducted in two phases: (1) measurement and characterization of the WTMD emissions and analysis of exposure, and (2) performing tests to expose the sample PMEDs to the emissions reproduced in the simulation system. The emissions measurements, simulation system, and PMED configurations are described below.

### Walk-through metal detector characterization

The WTMD magnetic field emissions from three WTMDs (designated A, B, and C) were characterized and spatially mapped using the commercial robotic positioner (DASY52 NEO system) interfaced with a commercial 3-axis magnetic field probe with frequency range covering of 30 Hz to 300 kHz (Electric Research and Management probe model 1850.001 with power supply model 1678.001).

**Fig. 1:**
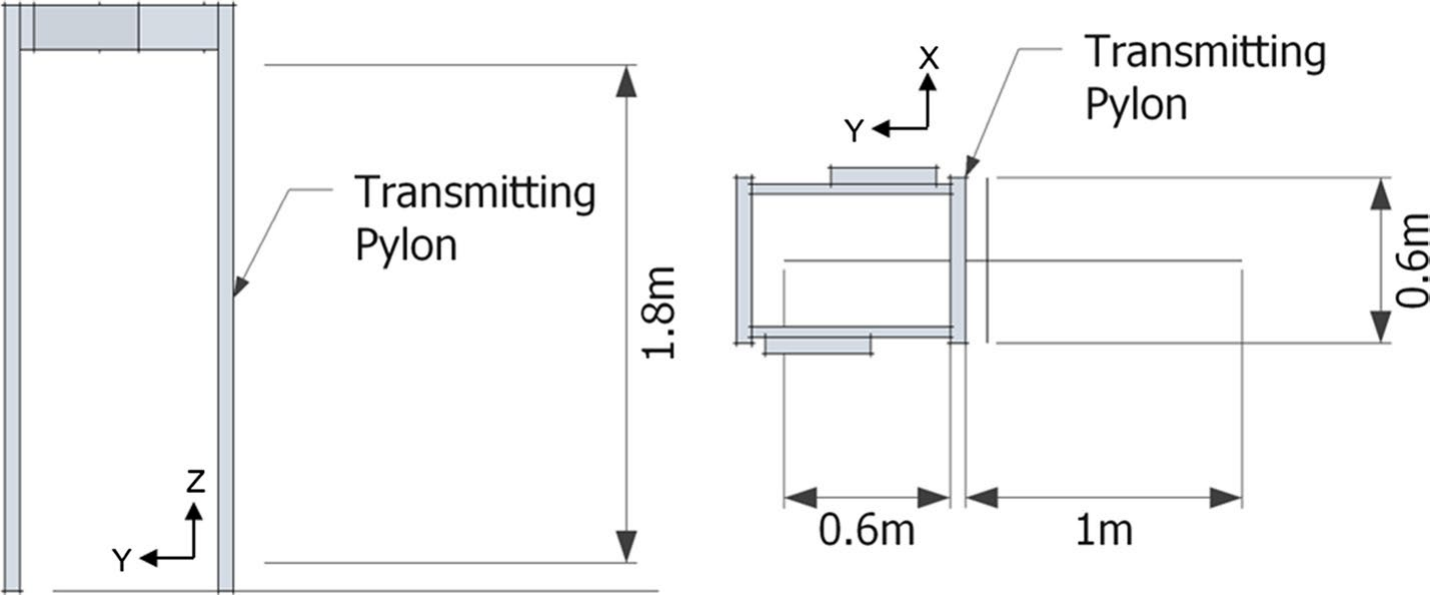
WTMD field mapping area. *Left* front view, *right* top view

The vector measurement of the magnetic field intensity was done in a number of planes parallel to the WTMD pylon (X-Z axis) that were positioned at different distances away from transmitting pylon (Y-axis) for both inside and outside of the WTMD. The entire field mapping area is shown in Fig. 1. The magnetic fields for each measurement planes (X-Z axis) were measured with 2.5 cm resolution. Along the Y-axis, the emissions fields were measured at 8 cm (the closest that the field probe could be positioned) and 10 cm then at 5 cm steps away from the WTMD transmitting pylon. On the inside of the WTMD, the magnetic field was measured up to 60 cm away from the transmitting pylon. On the outside of the WTMD, the measurement planes were measured up to 100 cm away from the transmitting pylon. All field strengths were measured in A/m root mean square (RMS).

The characterization of the WTMD waveforms were performed separately using oscilloscope (Lecroy LT264) and the 3-axis magnetic field probe. For each of the WTMDs, emission waveforms were captured and recorded at selected locations inside and outside of WTMDs including mid-way between pylons and 8 cm from inside and outside of transmitting pylon (Y-axis), and three different heights 25, 100, and 140 from the ground (Z-axis), and at center location on X-axis; a total of 9 points. These three heights were determined from anthropomorphic data as typical locations of the selected types of PMEDs [18].

### WTMD simulator system

A WTMD simulator system that was developed and validated in earlier studies of PMED EMI [5, 19] was used to allow for more controlled and consistent testing with emissions of WTMD. The design of the WTMD simulator was presented in [19] and earlier versions of the WTMD simulator were reported by Kainz et al. [5] and Seidman et al. [6]. The simulator system provides the capability to expose the sample PMEDs to field strengths above the maximum levels measured from the sample WTMDs, which range 21.3–27.9 A/m (see Table 4). This allows for extended exposure to help determine the threshold for EMI for the sample PMEDs. Further verification of WTMD simulator is described in “WTMD simulator verification” section.

**Fig. 2:**
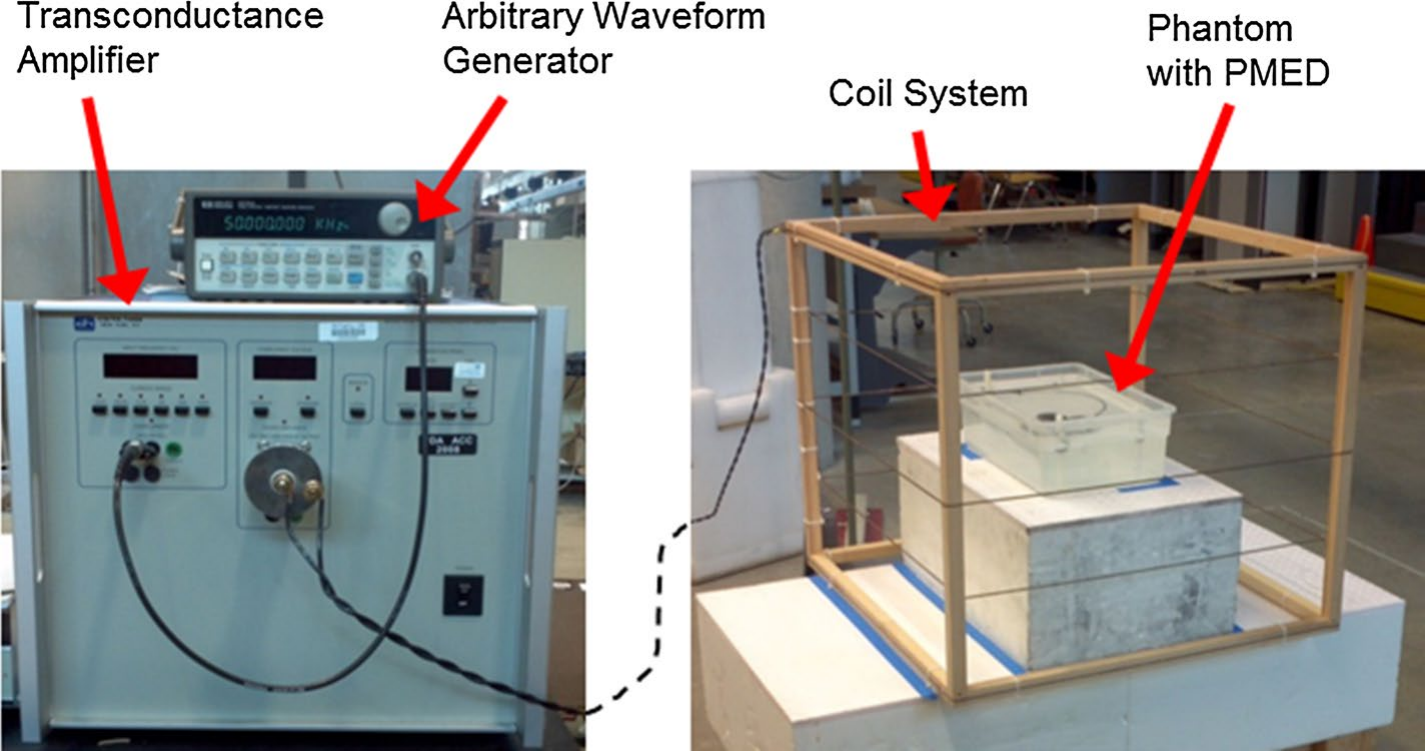
Example test setting with WTMD simulator

**Fig. 3:**
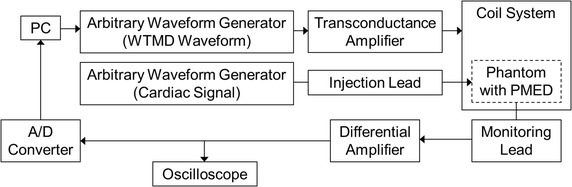
Block diagram of example test setting for cardiac devices

As illustrated in Figs. 2 and 3, the WTMD simulator system is built around a magnetic field coil (75 cm × 75 cm × 66 cm, 5-turn loop) driven with a commercial arbitrary waveform generator (Keysight, HP33120A) and a commercial transconductance amplifier with operating frequency of DC to 100 kHz (Clarke-Hess Model 8100). The recorded waveforms from WTMDs were loaded into the arbitrary waveform generator and used to generate the signal outputs that were injected into the magnetic field coil through the amplifier with necessary current to generate the targeted exposure field strength. For each simulated WTMD waveform, the magnetic field strength was measured over a 40 cm × 40 cm × 30 cm volume at the center of the simulator coil to ensure spatial uniformity of the exposure field. For each WTMD waveform, the difference between the maximum and minimum field strength measured inside the measurement volume was within −0, +2 dB (+25% field strength in A/m), where the minimum field strength measured inside the measurement volume was kept above the intended exposure field strength.

The PMEDs were exposed in the simulator to the magnetic fields created using the waveform from each WTMD. Seven specific waveforms were selected to represent all three WTMDs in the WTMD simulator. This is because the signals emitted from WTMD vary at different spatial locations, which makes it impractical to test for all possible waveforms from the WTMDs. However, the fields from the WTMD simulator are spatially uniform. As shown in Table 4, the signals emitted from WTMDs fall into two general types: continuous wave (CW) as seen from WTMD-A or pulsed as seen from WTMD-B and WTMD-C. WTMD-A emits CW signals from multiple transmitters within its transmitting pylon, and CW signals from each transmitter are dominated by one carrier frequency. Any of the variant waveforms measured and recorded at each spatial location would be a superposition of some combination of these different frequencies.

The waveforms selected for the simulator for WTMD-A are based on the spatial location 8 cm away from the transmitting pylon and either 25, 100, or 140 above the ground. The exposures waveforms at these three locations (designated as A1, A2, and A3 in Table 1) are intended to represent the patient exposures in proximity to transmitting pylon at typical heights where PMEDs are located in or on the patient’s body [18]. WTMD-B and WTMD-C emit pulsed signals with different waveforms present at different spatial locations. For simplification and to best manage the measurement and testing time, two different waveforms were chosen to represent the extreme cases for each of these pulsed WTMDs. The waveforms chosen include the most basic form of pulses (designated B1 for WTMD-B and C1 for WTMD-C) and the one consisting of multiple pulses with maximum number of pulses observed in the WTMDs (designated B2 for WTMD-B and C2 for WTMD-C). All sample PMEDs were exposed to the maximum field strengths possible from WTMD simulator for each waveform, as listed on Table 1 below.

**Table 1:** Maximum field strength of WTMD simulator generated for each waveform from each WTMD

WTMD source	Waveform	Maximum field strength generated in the WMTD simulator (A/m RMS)
WTMD-A	A1	22.9
	A2	80
	A3	79.7
WTMD-B	B1	24.9
	B2	45.9
WTMD-C	C1	30.3
	C2	74.9

For each waveform, the exposure magnetic field strength in RMS started at 1 A/m then next to 5 A/m and afterward incremented in 5 A/m steps up to the maximum field strength output of WTMD simulator. PMED exposure over the maximum field strength measured from actual WTMD was performed in order to investigate any potential EMI effects. The field strength was measured at a reference point at the lowest part of the phantom where minimum field strength was measured in order to ensure the entire phantom and device is exposed to the intended field strength or higher. This method assures the exposure is at least what was measured in sample WTMDs.

For each step, the exposure duration was 15 s while maintaining the exposure field uniformity. Observations of the PMEDs focused on looking for effects such as changes in waveform, amplitude, or rate. These could indicate malfunction, degradation of performance, or deviation beyond the tolerances indicated in the individual device specifications. If any effects on the sample PMEDs was observed, additional testing was performed with 1 A/m steps of increasing exposure starting at the field strength 5 A/m below where EMI effect was initially observed.

### Sample PMEDs and their configuration

The sample PMEDs were chosen to represent the typical medical devices that might present at the security checkpoint. These are not intended to provide a comprehensive array of all possible such medical device, but do reflect the types that are potentially susceptible to EMI from the lower frequency magnetic fields emitted by WTMDs [13] and reflect recent incident reports [15]. These devices were marketed devices at the time of the testing. The sample of PMED devices included 20 different devices:

- Six implantable cardiac pacemakers (P1–P6)
- Six implantable cardioverter defibrillators (ICDs) (D1–D6)
- Five implantable neurostimulators (N1–N5)
- Three body worn insulin pumps (I1–I3).

The configuration for testing of the sample PMEDs was based on previous work with these type devices and appropriate test standards. The implantable cardiac devices were tested under the settings of maximum sensitivity to the cardiac signals and bipolar configuration for both pacing and sensing of the lead. The maximum cardiac sensitivity setting for these devices was used because that setting was considered to represent the worst-case scenario for potential device susceptibility. This setting was used for both pacing mode and inhibited mode. The inhibited mode was achieved by injecting simulated cardiac signal as specified in the ISO 14117:2012 standard [20]. Five of the implantable cardiac pacemakers had unipolar settings, and those devices were also set and tested in this mode for both pacing and sensing. The unipolar lead configuration was tested when it was possible because it is generally considered more susceptible than bipolar configuration [21]. If an EMI effect was observed while the PMED setting was in maximum sensitivity, the PMED was re-tested using its nominal sensitivity setting in order to further assess potential device effects. As a safety precaution all ICDs were programmed to have the defibrillation shock therapy output off while still set in the monitoring mode. This was not possible for three ICDs, and the amplitude of the potential output shock was set to its lowest value for those ICDs. An overview of the device settings such as sensitivity and lead configuration for the implantable cardiac devices is illustrated in Tables 2 and 3 below.

**Table 2:** Implantable cardiac pacemaker device setting

Device	Mode	Lead configuration	Sensitivity range (maximum)	Sensitivity range (nominal)
P1	DDD	Bipolar/unipolar	Atrial: 0.1–0.6 mV Ventricle: 0.2–3 mV	Atrial: 0.4–1 mV Ventricle: 1–3 mV
P2	VVI	Bipolar/unipolar		
P3	VVI	Bipolar/unipolar		
P4	DDD	Bipolar/unipolar		
P5	DDD	Bipolar		
P6	DDD	Bipolar/unipolar

**Table 3:** Implantable cardioverter defibrillator device setting

Device	Mode	Lead configuration	Sensitivity range (maximum)
D1	DDD	Bipolar	Atrial: 0.1–0.3 mV Ventricle: 0.1–0.3 mV
D2	VVI	Bipolar	
D3	DDD	Bipolar	
D4	DDD	Bipolar	
D5	DDD	Bipolar	
D6	VVI	Bipolar	

The implantable neurostimulator devices were configured and tested in bipolar mode. The unipolar mode was available for two implantable neurostimulator devices, and those devices were also configured in this mode. These PMEDs were configured to provide a continuous output so that the device continuously delivered the programmed stimulation output. The implantable neurostimulators were also exposed in the simulator with the stimulation output turned off in order to assess any potential unintended stimulation.

The insulin pump PMEDs were configured and tested in the idle (standby) mode to determine a baseline, and the bolus delivery mode. The bolus delivery mode was tested for device performance in delivering proper therapy. In addition, the device was tested in alarm mode where the device alarm was triggered prior to the exposure. Idle and alarm modes were tested for possible mode change or unintended delivery of therapy.

The general approach for testing each PMED was to expose each sample to simulated field in the WTMD simulator using the following steps:

1. Check and program the PMED settings prior to any exposure testing,
2. Perform the exposure testing, monitor and record the device electrical output (active implantable devices), and
3. Perform post exposure checks on the PMED.

The implantable cardiac PMEDs were individually placed in a saline-filled tank “phantom” in the WTMD simulator coil. PMED was placed on a plastic grid to ensure each PMEDs were properly configured and positioned at the same location inside the phantom. The configurations for the implantable pacemaker and ICD leads were based on the test method in the ANSI/AAMI/ISO 14117:2012 standard [20]. These PMEDs were placed in a saline solution (equivalent to 375 Ω cm) with the leads configured to have lead loop area of 225 cm^2^ with the plane of the lead loop perpendicular to the H-field created inside the WTMD simulator. Per ANSI/AAMI/ISO 14117:2012 standard [20], the saline solution used is intended to represent electrical property of human body tissue.

The implantable neurostimulator PMEDs were configured according to ANSI/AAMI/ISO 14708-3:2008 standard [22] with the sample submerged in a phantom tank with saline solution (equivalent to 375 Ω cm). The PMED leads were placed in a spiral configuration with the plane of the loop surface perpendicular to the H-field created inside the WTMD simulator coil. The distance (D) between the center of the neurostimulator device’s pulse generator and the tip was determined using the following equation based on the ANSI/AAMI/ISO 14708-3:2008 standard, where L is the lead length.

$$D = \sqrt {(0.09 \times L^{2} )/\pi }$$

The insulin pump samples were placed on a low dielectric Styrofoam block at the center of the simulator coil. Two of the three insulin pumps had the capability to be coupled with a glucose sensor which was placed within the coil 3.5 cm away from the insulin pumps while actively transmitting simulated glucose measurements. The glucose sensor provided consistent device communications with the insulin pump. The insulin pump devices do not have a direct electrical output which makes these device more difficult to monitor. To accomplish monitoring of the insulin pumps two small coils (8 turn 3.5 cm diameter loop antennas) were constructed and combined to couple to the emissions from the pump motor when it is activated to release the insulin into the patient. The output from these coils were connected to a differential amplifier to provide adequate signal to noise ratio and connected to an oscilloscope.

### WTMD simulator system verification

An additional verification testing was performed by exposing a PMED to an actual sample WTMD. This was done to assure the WTMD simulator testing provides a reasonable representation PMED exposure that is expected in a typical WTMD.

## Results

### Emission characteristic

**Fig. 4:**
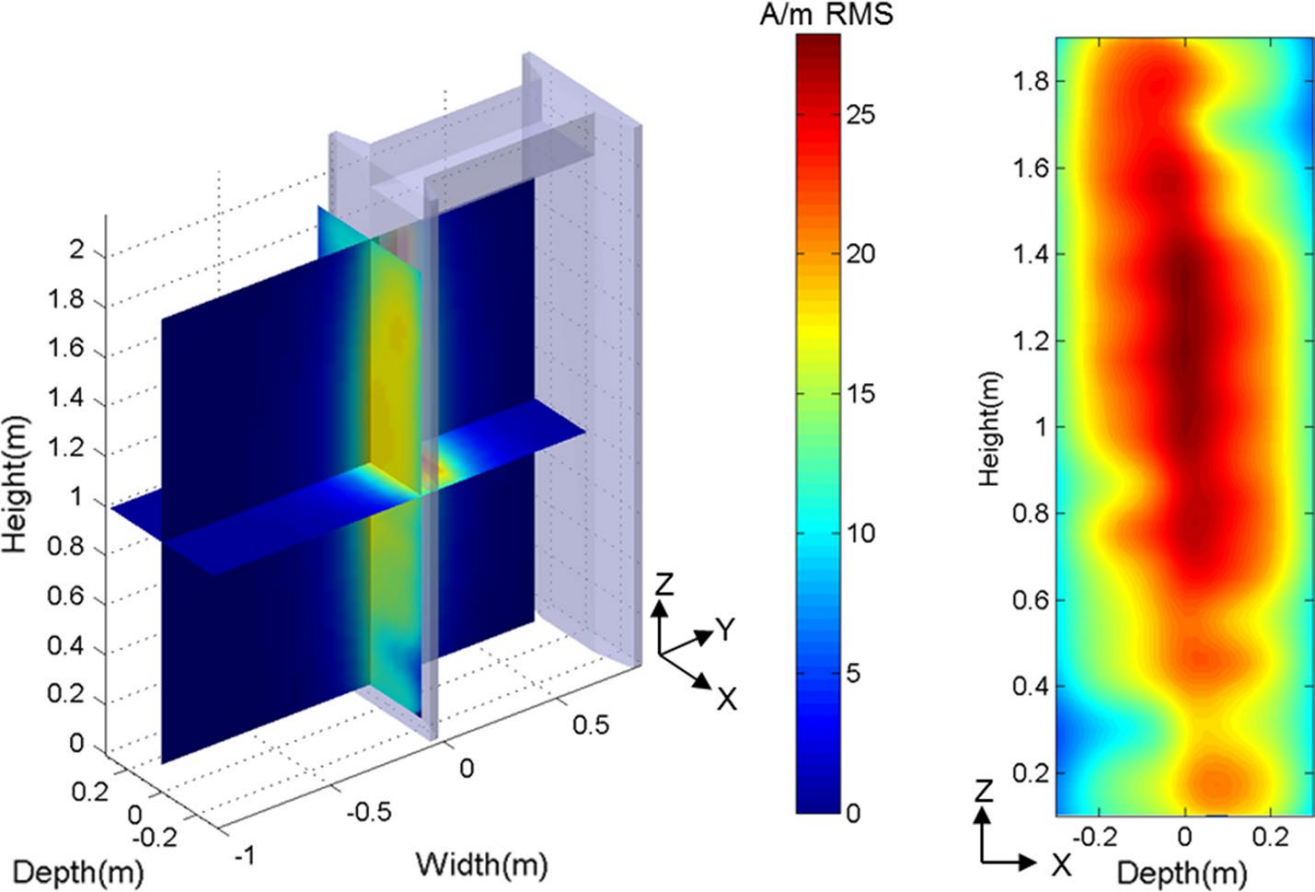
Example magnetic field emission measurement from WTMD-A (A/m RMS). Magnetic field value of vector magnitude over entire mapping area. *Left* three slices represent center vertical (Y–Z) plane, 1 m height horizontal (X–Y) plane, and vertical (X–Z) plane parallel and 8 cm outside away from the transmitting pylon. *Right* vertical (X–Z) plane parallel and 8 cm inside away from the transmitting pylon

A summary of the WTMDs’ emission characteristic is presented in Table 4. All field strengths were measured in A/m RMS and the last column in Table 4 shows the absolute maximum field strength measured in each WTMD. The absolute maximum field strength is the highest vector magnitude field strength measured throughout entire field mapping area. In addition to the maximum field strength for each WTMD another metric called averaged maximum field was developed. This is calculated by averaging the field measurement of a single field component that is normal to the transmitting pylon (Y-axis) over the device area of each PMED. For implantable pacemakers, ICDs, and neurostimulators this area includes the area encompassed by the device leads. An example of magnetic field emission measurement is shown in Fig. 4.

**Table 4:** Magnetic field characteristics from the sample WTMDs

WTMD	Waveform	Frequency/pulse repetition rate	Pulse width (µS)	Rise/fall time (µS)	Absolute maximum field measured (A/m RMS)
WTMD-A	Multiple CW	3–14 kHz	N/A	N/A	21.3
WTMD-B	Pulsed	200–250 Hz	100–200	5–50	21.1
WTMD-C					27.9

### Electromagnetic interference observations

EMI effects were observed on six of the 20 PMEDs when they were exposed to the maximum field strength generated by WTMD simulator. Five of the PMEDs that showed EMI effects were implantable cardiac devices and one of the PMEDs was an implantable neurostimulator. Table 5 lists the types of EMI effects observed for five implantable cardiac and one implantable neurostimulator devices. All PMEDs returned to normal operation once removed from the WTMD simulator exposure field with no lasting effects. None of the ICD and insulin pumps devices indicated EMI effects.

**Table 5:** EMI observed during the testing

Device type	EMI observed
Implantable cardiac pacemakers	Pacing inhibition
	Pacing induced
	Pacing rate change
	Atrial/ventricular pacing interval changes
Implantable neurostimulators	Pulse inhibition
	Pulse rate changes
	Pulse waveform changes
Implantable cardioverter defibrillators (ICDs)	No EMI
Body worn insulin pumps	No EMI

**Fig. 5:**
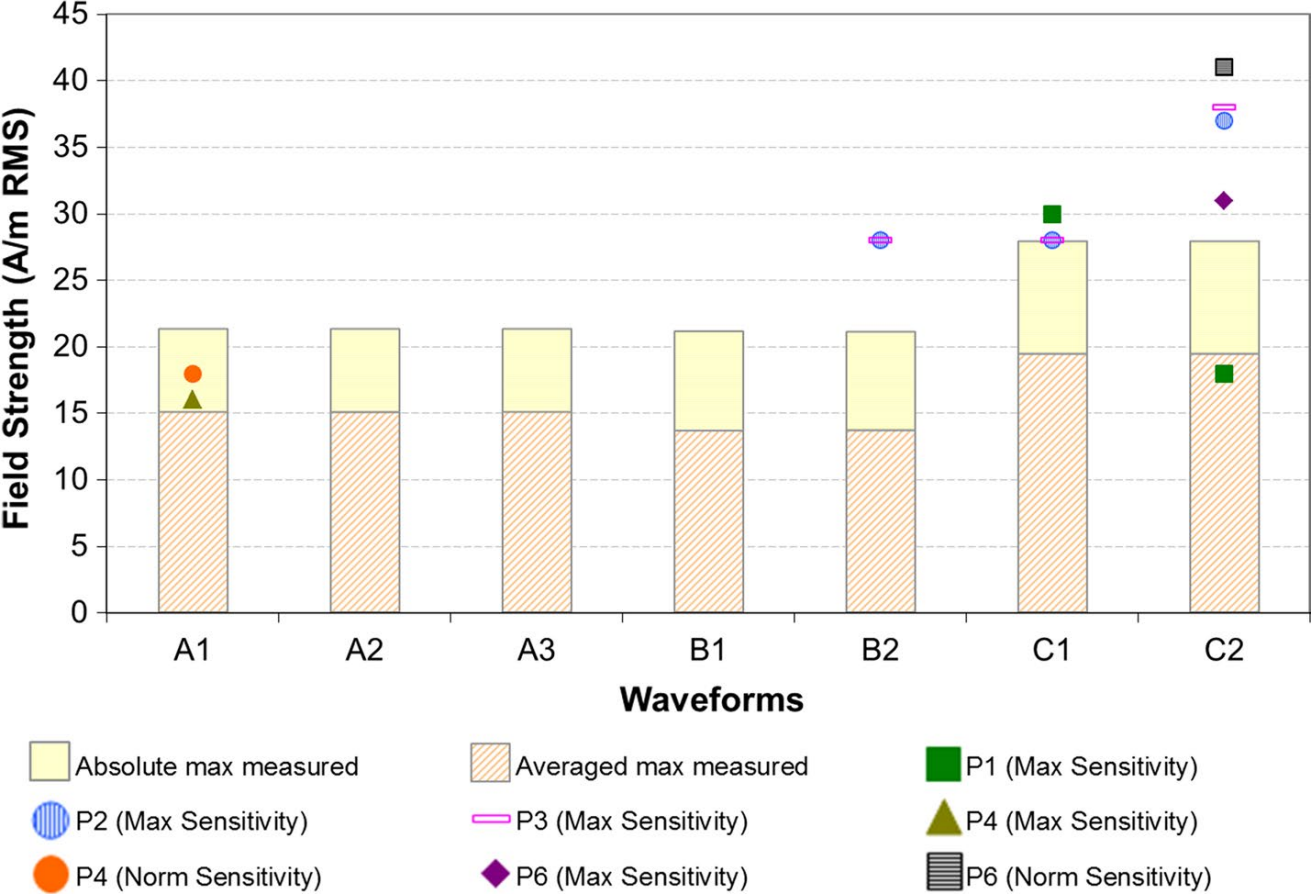
*Scatter points* are the threshold for EMI (if observed) for six implantable cardiac pacemakers (P1–P6) tested across the seven different waveforms in WTMD simulator. For each waveform, PMEDs were exposed to maximum field generated in WTMD simulator (see Table 1) in order to investigate any potential EMI. Bars represent measured exposure field strengths in actual WTMDs. The “averaged max measured” fields are averaged over a pacemaker lead loop area of 225 cm^2^

For the implantable cardiac PMEDs tested, the observed effects included pacing inhibition, induced pacing, pacing rate change, and atrial and ventricular interval change, when these devices were set to their maximum sensitivity and unipolar configuration. Using nominal cardiac sensitivity, two of the five devices revealed similar EMI effects as were observed when the devices were set in maximum sensitivity and unipolar configuration. However, the EMI effects for these two devices using nominal cardiac sensitivity were observed at 12–33% higher exposure field threshold than when those devices were set in maximum cardiac sensitivity. Thus, the devices were less susceptible with nominal sensitivity than with maximum sensitivity. No EMI was observed for the implantable pacemakers in bipolar configuration. The exposure field thresholds for EMI for implantable pacemakers are shown in Fig. 5.

**Fig. 6:**
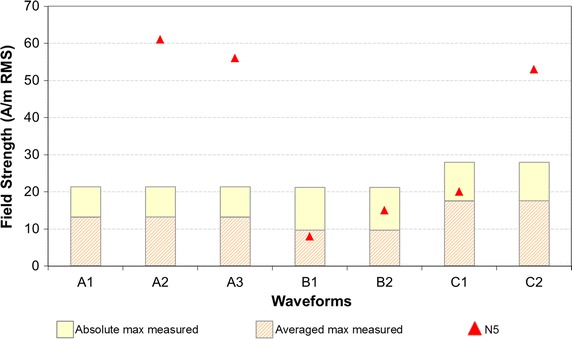
*Scatter points* in *red* are the threshold for EMI for the implantable neurostimulator (N5) tested across the seven different waveforms in WTMD simulator. For each waveform, PMEDs were exposed to maximum field generated in WTMD simulator (see Table 1) in order to investigate any potential EMI. *Bars* represent measured exposure field strengths in actual WTMDs. The “averaged max measured” fields are averaged over the neurostimulator lead loop area

For the implantable neurostimulators PMEDs tested, EMI effects were observed in one out of the six devices in the WTMD simulator. The effects included pulse inhibition, pulse rate change, and pulse waveform change. The exposure field thresholds for EMI for the implantable neurostimulator are shown in Fig. 6.

### WTMD simulator verification

The WTMD simulator test results for a PMED were verified by exposing PMED N5 (implantable neurostimulator) to the emissions from an actual WTMD (WTMD-B). PMED N5 was used for verification because the observed EMI occurred generally at a lower field strength than other PMEDs. For this testing, a vertical saline-filled phantom was used with the plane of the PMED lead loop surface parallel to the transmitting pylon. EMI effects were observed when the surface of the phantom was separated from the transmitting pylon by less than 10 cm. At this location, the averaged field strength over the loop area was 9.6 A/m. The test with PMED N5 was repeated in WTMD-B to confirm the EMI observations. The exposure field strength thresholds that produced the same effect in the WTMD simulator were 8 A/m for WTMD-B waveform B1 and 15 A/m for waveform B2. The field strength threshold from actual WTMD-B falls between the thresholds observed from waveform B1 and B2 in the WTMD simulator. This indicates the WTMD simulator provides a reasonable representation of the range of waveforms emitted by WTMD-B, and reinforces previous studies [5, 6] showing the WTMD simulator is a reasonable facsimile for assessing PMED susceptibility to EMI.

## Discussion

The results of this study suggest that exposure to the emissions from WTMDs can cause EMI effects in certain more contemporary PMEDs. In this study EMI effects were observed in pacemakers P1 (maximum sensitivity) and P4 (both maximum and nominal sensitivity), and neurostimulator N5, during WTMD simulator exposure below the typical exposure field strength measured in the sample WTMDs. Large portion of the EMI effects were observed at exposure field strength higher than the maximum field strength measured from actual WTMD. Further, certain implantable cardiac devices were more susceptible to pulsed signals when set into the unipolar configuration. The likelihood of EMI effects related to WTMD exposure was lower for bipolar lead configuration. This appears to be due to increased induced voltage resulted from larger effective antenna area formed by unipolar lead configuration [21, 23].

Assessing clinical significance of EMI effects observed during the testing was outside the scope of the present study. However, all observed EMI effects were transient and the PMEDs returned to normal operation within a few seconds after removal from the exposure magnetic field. Nonetheless, this study shows EMI effects on the sample PMEDs such as pacing inhibition might pose risks to patients who are dependent on the PMED. In addition, the device effects such as charge imbalance caused by the distorted waveform that was observed for implantable neurostimulator might result in unintentional stimulation [6].

The measurements of the fields in and around the WTMDs illustrated in Fig. 4 shows that these fields decrease rapidly with distance away from transmitting pylon. One way to reduce the risk of the EMI is to increase the separation distance between the PMED and transmitting pylon and move away after the security scan. Measurements from the three WTMDs indicate the averaged maximum field strength was reduced to 3 A/m or less for all WTMDs at 30 cm away from transmitting pylon. Another way to reduce the risk is by minimizing the exposure time. This can be accomplished by proceeding through the WTMD at a regular or brisk pace to reduce exposure time. This is consistent with the recommended practice for patients with PMEDs around WTMD [13, 14]. This would minimize probability of EMI considering proximity to the WTMD needed for exposure to field strength where EMI was observed in this study.

### Study limitations

Although this study is limited to the small number of sample WTMDs, these samples WTMDs are typical of the products that are deployed across public buildings, airports, and other locations. There is information based on adverse events reported in the FDA’s MAUDE database and previous work that indicate other types of security systems such as anti-theft or electronic article surveillance (EAS) systems and hand-held metal detectors (HHMDs) could interfere with PMED [1–4, 11, 13, 15]. Thus, additional investigations for EAS and other security systems as well as newer types of WTMD are needed to further understand the potential susceptibility of PMEDs to the emissions from various security systems that are increasingly common in the environments where medical devices are used and patients are exposed.

In addition, the present study is limited to the emissions waveforms that were captured at a limited number of locations in and around WTMDs. While the WTMD simulator provides reasonable representations of actual WTMD, the waveforms were selected based on the probable location of the PMED in and around WTMD, which given the array of potential locations, might not represent the worst-case exposure for the PMEDs. Further studies are needed to more fully evaluate the WTMD simulator’s ability to reproduce the WTMD fields for a larger array of cases and to identify and characterize representative worst-case exposure and testing conditions.

## Conclusions

The potential susceptibility to EMI effects in a selected group of contemporary sample PMEDs was assessed for locations in and around three WTMDs. Exposure tests of the PMEDs were performed using the FDA designed WTMD simulator recreate exposures based on measurements of the emissions from the sample WTMD systems. Transient EMI effects were observed for two implantable cardiac pacemakers and one implantable neurostimulator exposed to the fields created in the simulator that corresponds to field strength from actual WTMD. The affected PMEDs returned to pre-exposure condition without user intervention within a few seconds after removal from the simulated exposure fields. Because the observed effects were transitory and with no lasting effects on the PMEDs, it appears the risks associated with PMED EMI related to WTMD exposure could likely be mitigated by taking precautions such as appropriate separation distance and limiting exposure time. PMED patients and manufactures should be aware of the potential susceptibilities associated with WTMD exposure and mitigate potential risks for EMI including limiting exposure and exposure time. These findings are consistent with earlier studies and the recommendations presented by FDA and others [5, 6, 13, 14]. Further investigation is needed to evaluate and assess EMC among PMEDs across a broader range of security systems including HHMDs and EAS systems.

## Abbreviations

EM: electromagnetic
EMC: electromagnetic compatibility
EMI: electromagnetic interference
EAS: electronic article surveillance
HHMD: hand-held metal detectors
ICD: implantable cardioverter defibrillators
MAUDE: manufacturer and user facility device experience
PMED: personal medical electronic devices
WTMD: walk-through metal detector
